# Persistent Homology for the Quantitative Evaluation of Architectural Features in Prostate Cancer Histology

**DOI:** 10.1038/s41598-018-36798-y

**Published:** 2019-02-04

**Authors:** Peter Lawson, Andrew B. Sholl, J. Quincy Brown, Brittany Terese Fasy, Carola Wenk

**Affiliations:** 1 Department of Biomedical Engineering, Tulane University, New Orleans, Louisiana 70118 USA; 2 Department of Pathology and Laboratory Medicine, Tulane University School of Medicine, New Orleans, Louisiana 70118 USA; 3 Department of Computer Science, Tulane University, New Orleans, Louisiana 70118 USA; 4 School of Computing and Department of Mathematical Sciences, Montana State University, Bozeman, Montana 59717 USA

## Abstract

The current system for evaluating prostate cancer architecture is the Gleason grading system which divides the morphology of cancer into five distinct architectural patterns, labeled 1 to 5 in increasing levels of cancer aggressiveness, and generates a score by summing the labels of the two most dominant patterns. The Gleason score is currently the most powerful prognostic predictor of patient outcomes; however, it suffers from problems in reproducibility and consistency due to the high intra-observer and inter-observer variability amongst pathologists. In addition, the Gleason system lacks the granularity to address potentially prognostic architectural features beyond Gleason patterns. We evaluate prostate cancer for architectural subtypes using techniques from topological data analysis applied to prostate cancer glandular architecture. In this work we demonstrate the use of persistent homology to capture architectural features independently of Gleason patterns. Specifically, using persistent homology, we compute topological representations of purely graded prostate cancer histopathology images of Gleason patterns 3,4 and 5, and show that persistent homology is capable of clustering prostate cancer histology into architectural groups through a ranked persistence vector. Our results indicate the ability of persistent homology to cluster prostate cancer histopathology images into unique groups with dominant architectural patterns consistent with the continuum of Gleason patterns. In addition, of particular interest, is the sensitivity of persistent homology to identify specific sub-architectural groups within single Gleason patterns, suggesting that persistent homology could represent a robust quantification method for prostate cancer architecture with higher granularity than the existing semi-quantitative measures. The capability of these topological representations to segregate prostate cancer by architecture makes them an ideal candidate for use as inputs to future machine learning approaches with the intent of augmenting traditional approaches with topological features for improved diagnosis and prognosis.

## Introduction

**Figure 1:**
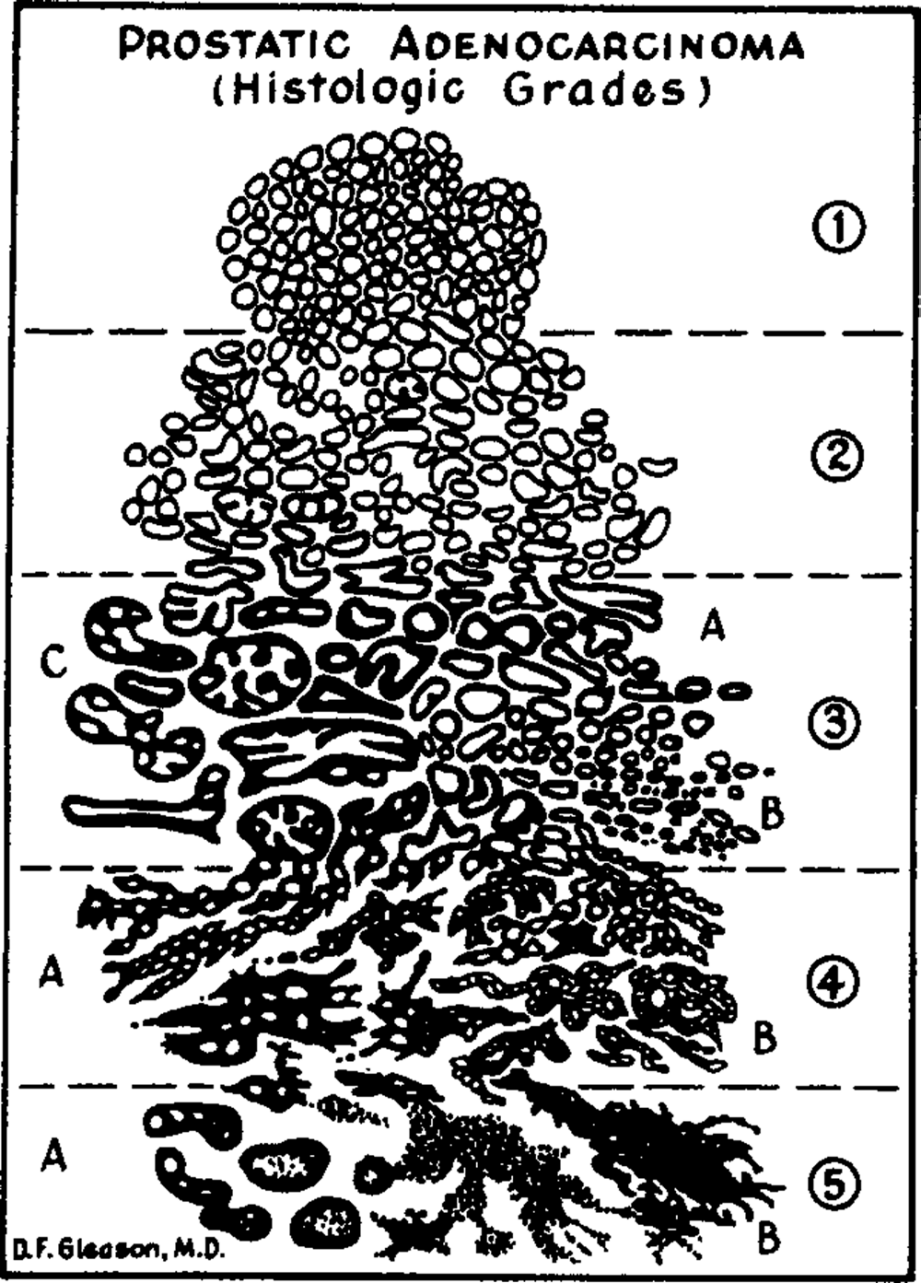
Original Gleason grading system drawing. Gleason drawing reprinted from the Veterans Administration Cooperative Urological Research Group.

Prostate cancer (PCa) is the second most common cancer in men worldwide with an estimated 1.1 million cases diagnosed in 2012^1,2^. In addition, PCa corresponds to the fifth leading cause of cancer deaths in men worldwide, and the third leading cause within the United States^2,3^. To make a diagnosis, a trained pathologist evaluates the tissue microarchitecture on hematoxylin and eosin (H&E) stained slides of a prostate biopsy and grades it based on the two most dominant malignant glandular patterns present. The most widely used histological classification scheme is the Gleason grading system. While the Gleason grading system currently remains the most powerful predictor of prognostic outcome in PCa, it suffers from high variability due to the subjective nature of grading, either by comparison to drawings (see Fig. 1) or example digital images representing common architectural patterns. Even within Gleason patterns, there are differing architectures that the Gleason system fails to account for, each of which may have their own prognostic significance. A clear need exists for a truly quantitative and reproducible method of histological classification of prostatic adenocarcinoma at a finer granularity than existing systems. With advances in computation there is an opportunity to discover new architectural groups based on objective, quantitative methods of architecture assessment. Such an approach could discover new image biomarkers that would inform pathology and could be used as inputs to appropriate computer aided diagnostic (CAD) systems.

Topological Data Analysis (TDA) is uniquely suited to the problem of quantifying the architecture of prostate cancer. The Gleason grading system relies exclusively on recognition of the architectural patterns formed by arrangements of carcinoma cells and adjacent stroma for histological classification. TDA provides a means of understanding the shape of data, and thus architecture, even in higher dimensional space. Persistent homology, a subset of TDA, represents a unique way to characterize PCa histological architecture across a range of scales, capturing features ranging from individual cell organization to glandular shape and inter-glandular arrangement. Persistent homology has been successfully applied to breast cancer histology images to distinguish breast cancer genetic subtypes^4^. However, the application of persistent homology for the characterization of PCa architecture has not been previously described. We are applying persistent homology to the study of prostate cancer architecture and its relationship to traditional histopathology in the context of Gleason patterns. The goal of this study is to evaluate whether persistent homology could be used to describe PCa architecture in a way that can not only correlate with the Gleason pattern, but provide additional insights into PCa architecture at a level of granularity beyond the Gleason system.

## Background and Methodology

### Prostate Cancer Architecture

**Figure 2:**
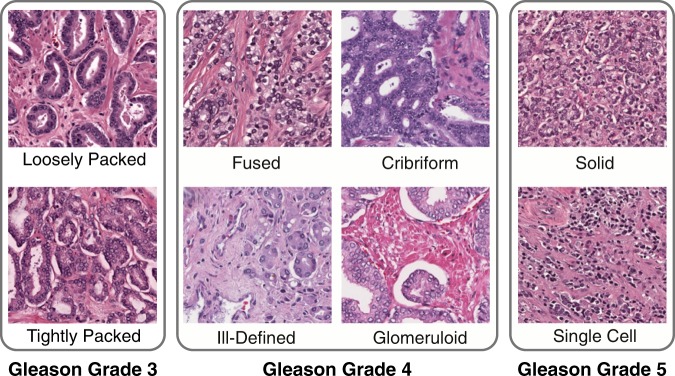
Key Common Architectures. Gleason grade 3 corresponds to Gleason 3 glandular patterns, both loosely packed, characterized by well-circumscribed glands, and crowded glands, characterized by minimal stromal space between glands. Gleason grade 4 corresponds to four common Gleason 4 patterns, fused, cribriform, ill-defined, and glomeruloid glands. Gleason grade 5 corresponds to two common Gleason 5 patterns, single-cell infiltrating and solid cell types.

The most widely accepted method of evaluating PCa architecture is the Gleason grading system, shown in Fig. 1. When PCa is identified in H&E-stained histology slides, the Gleason grading system is employed to stratify the PCa into groups of increasing aggressiveness by architectural differences alone. The Gleason score is comprised of five architectural Gleason *patterns*, arranged in increasing degrees of neoplastic differentiation, corresponding to increasingly poor prognostic outcomes^5^. Each of these patterns has a value assigned to it, called a *grade*, ranking the aggressiveness of the prostate cancer from 1 (least) to 5 (most). A Gleason *score* is then assigned by summing the the most prevalent (e.g., Gleason 4) and second most prevalent (e.g., Gleason 3) patterns corresponding to the reference drawing, yielding a score (e.g, Gleason score: 4 + 3 = 7). This score serves to aid in the stratification of patients into prognostic or treatment groups, with patients with a score of 6 or less generally receiving more conservative treatment, while those with a score of 7 or above receiving more aggressive treatment. While the Gleason grading system currently remains the most powerful predictor of prognostic outcome in PCa, it suffers from high intra and inter-observer variability due to the subjective nature of the grading scheme^6–12^. In a review of grading reliability in PCa, intra-observer agreement on Gleason grade was reported in only 43–78% of cases^12^. In addition, inter-observer variability was high with concordance between 50–60% in most cases^12^. The Gleason grading system also lacks granularity. In some cases, such as the NCCN nomogram, only the overall score is considered, despite an understanding that, for example, 3 + 4 = 7 and 4 + 3 = 7 scores are prognostically distinct, and it has been shown that 4 + 3 = 7 has significantly worse prognostic outcomes^13^. Increasing prevalence of Gleason pattern 4 is associated with an increased risk of biochemical recurrence and cancer specific mortality. Prognostically, a patient’s outcome is intermediate between the two scores, evidence that Gleason scores should be considered as a continuum, not as discrete scores^14^. This failure, in part, of the Gleason grading system to stratify by the relative contribution of Gleason patterns has resulted in the development of a new grading system, Grade Groups, adopted in the 2014 International Society of Urological Pathology (ISUP) Consensus Conference on Gleason Grading of Prostatic Carcinoma^15^. Grade Groups allow for more accurate Gleason pattern stratification by treating 3 + 4 as prognostically distinct from 4 + 3. In a follow-up validation study, after radical prostatectomy, Gleason Grade Group 2 (3 + 4) patients had a four year biochemical recurrence (BCR) free rate of 74% whereas Gleason Grade Group 3 (4 + 3) patients had a 63% BCR free rate^16^. The difference in BCR free rates by Grade Group is evidence that further stratification by relative contribution of architectural subtypes is necessary to better correlate to patient prognostic outcomes. Grade Groups are limited to primary and secondary Gleason patterns and do not account for tertiary Gleason patterns which have been shown to have a risk of recurrence that lies between existing Gleason scores^17^. While Grade Groups have addressed problems associated with insufficient granularity in the Gleason score, they cannot address the inherent variability in reviewers due to the subjective nature of the pattern evaluation. In addition, some studies have indicated varying prognostic outcomes for the architectural subtypes that exist within single Gleason patterns, as shown in Fig. 2. Work by Dong *et al*.^18^ indicated that within patients with Gleason 4 PCa, the dominant architectural patterns of Gleason 4 that are present (i.e., poorly formed, fused, or cribriform) is associated with significantly different prognostic outcomes, with patients exhibiting all architectures having poorer BCR free rates than those exhibiting any one pattern (66% vs. 76%). In addition, the presence of cribriform pattern in Gleason 4 patients was correlated to lowered BCR free rates, whereas the presence of fused pattern was associated with higher BCR free rates in Gleason 4 patients^19^. Grade Groups and Gleason scores are not mutually exclusive, but instead are often used in conjunction. The International Collaboration on Cancer Reporting (ICCR) official guidelines require the reporting of both Grade Groups 1–5 (or ISUP Grade 1–5 in ICCR notation) and Gleason score. Reporting of both Grade Groups and Gleason score is also accepted practice at Tulane Medical Center. Despite the use of both Grade Groups (ISUP Grades) and Gleason scores, an increase in granularity of grading architectural patterns may be better predictive of patient outcomes^20^.

### Topological Data Analysis

Persistent homology (PH), a tool from topological data analysis (TDA), serves as the basis for this work. In general, TDA comprises a series of methods and tools for evaluating qualitative geometric and topological properties of data. PH evaluates the “shape” of data, as captured by connected components, loops, voids, and other higher-dimensional features that exist within the data at varying scales. The *features* that we are interested in are the *homology groups*, which are an algebraic representation of those components, tunnels, and holes. We use *persistent* homology, which allows us to study these features at varying scales. We provide a concise definition of persistent homology (PH) and persistence diagrams (PD) below; but we refer the reader to Edelsbrunner and Harer^21^ and references therein for further details.

#### Persistent Homology

**Figure 3:**
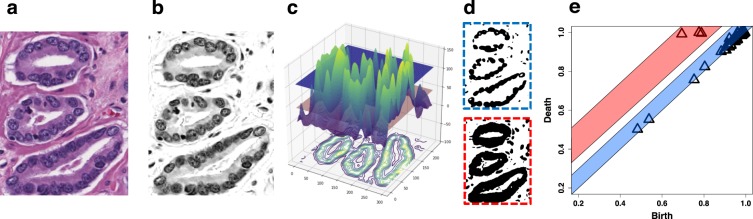
Generating a persistence diagram by computing PH on pixel intensities for an H&E image. (**a**) Three well circumscribed glands of an example Gleason 3 pattern histopathology image. (**b**) The same Gleason 3 pattern after color deconvolution isolates the hematoxylin color channel. (**c**) A 3D surface plot of the super-level set of pixel intensities of the Gleason 3 pattern image. Pixel intensities *i* of the image *I* are inverted as *max*_*i*_(*I*) − *i*, and a Gaussian blur applied for noise reduction for clarity of visualization, with the highest peaks corresponding to nuclei. A contour plot of the pixel intensities *i* is projected on the x-y plane of the surface plot. Two x-y plane slices of the surface plot are shown, with the red plane corresponding to *i* = 45, and the blue plane corresponding to *i* = 106. (**d**) Two thresholded binarizations of the deconvolved image. The binarization with the blue dashed line corresponds to smaller components captured by the filtration at the position of the blue plane in (**c**). The binarization with the red dashed line corresponds to the filtration at the red plane, where the components have merged to form three cycles, which correspond to the glands in (**a**). (**e**) The persistence diagram computed on the deconvolved image with the black triangles corresponding to cycles (one-dimensional topological features). The blue and red bands represent features present at the blue and red plane, respectively, in (**c**) and shown as thresholded images in (**d**).

Fig. 3 is an illustrative example that shows the steps of computing persistent homology on a simple three gland example. In Fig. 3b, we see three ellipsoidal structures formed by arrangements of cells, corresponding to three prostate glands. In Fig. 3d, super-level sets of pixel intensities captures these ellipsoids; that is, for a given arbitrary threshold *τ* (two of which are shown in Fig. 3d), we define the super-level set: *L*_*τ*_ = {*x*: *f*(*x*) ≥ *τ*}, where *f*: *I* → [0,255] is the function of pixel intensities for an 8-bit image. Looking closely, we notice that many gaps exist in the gland structures for the threshold chosen for the top of Fig. 3d, and extra information (additional connected components) are captured using the threshold in the bottom figure. In other words, a single threshold is not sufficient. *Persistent homology* tracks the changes in homology (i.e., the topological features that we are interested in) over a range of thresholds. More concretely, we follow the evolution of sub-level sets through decreasing values of *τ*:

1 $${L}_{255}\subseteq {L}_{254}\subseteq \cdots \subseteq {L}_{1}\subseteq {L}_{0}=I\mathrm{.}$$

This sequence of nested topological spaces is called a *filtration*, and induces a sequence of vector spaces (the homology groups) connected with linear transformations for each integer *k* ≥ 0:

2 $${H}_{k}({L}_{255})\to {H}_{k}({L}_{254})\to \cdots \to {H}_{k}({L}_{1})\to H({L}_{0})\mathrm{.}$$

Although persistent homology is a very general tool, we use ${{\mathbb{Z}}}_{2}$-coefficients (i.e., we do arithmetic modulo two). We define *β*_*k*_ to be the rank of *H*_*k*_. For *k* = 0, the homology group *H*_0_ (zero-dimensional features) has one generator per connected component. Additionally, *β*_1_ counts the number of loops or tunnels (one-dimensional features) and *β*_2_ counts the number of voids (two-dimensional features). Persistent homology tracks creation and merging of generating classes in *H*_*k*_(*L*_*τ*_) as the height threshold *τ* decreases through the filtration. In sum, persistent homology tracks topological features, and labels each with an interval of existence.

#### Persistence Diagram

Topological features that persist through a filtration can be plotted as a *persistence diagram*, which comprises a plot of all homology features, by class, as persistence pairs (*p*, *q*) that appear, or are “born” at step *p* in the filtration, and are destroyed or “die” at step *q* in the filtration. Thus, the length of *q* − *p* corresponds to the lifespan of a homology class feature in the filtration, and when plotted with “birth” on the x-axis and “death” on the y-axis, the persistence corresponds to the vertical distance from the diagonal. In the example of the three glands in Fig. 3, the three longest one-dimensional features, circles or loops, correspond to the three cycles of the three glands. Thus, information about the importance of features is encoded in the scale of persistent features. Here, smaller cycles can be attributed to the arrangement of cells, while the largest persistent features (those with the furthest off-axis distance) serve to capture the higher level arrangement underlying the structure of the three glands.

#### Application of Persistent Homology to Prostate Cancer

PCa architecture is comprised of 3D glands and acini, surrounded by a fibromuscular stroma that, in a 2D histological slice, correspond to rings of nuclei surrounding intraluminal space, and circumscribed by stroma. The size, number, arrangement, and integrity of these glands correspond to the level of aggressiveness of the PCa according to the Gleason score. The architectural nature of PCa features makes them highly suited to characterization by TDA. Fig. 3 shows an example of PH computed on a simple three-gland Gleason 3 image. Fig. 3b illustrates the hematoxylin channel, a nuclear specific color channel extracted computationally through color deconvolution, explained in more detail in the materials and methods. This computation of a grayscale representation of the hematoxylin color channel can be considered as a level set function over which we can generate a filtration and compute persistence. For the purposes of visualization, the pixel intensity is inverted, so in Fig. 3c the peaks of the surface plot are areas of maximal intensity corresponding to nuclei, represented by a super-level set. By panning a plane through the superlevel set from top to bottom and performing a global binarization of the image at each intensity value of the panning plot, an ordered sequence of growing superlevel sets is generated, known as a *filtration*. As the plane pans from top to bottom it first captures a series of smaller cycles corresponding to the many peaks of the nuclei, as shown in the thresholded binarizations at the pixel intensity indicated by the blue plane and shown in the blue outlined image in Fig. 3d. As the plane pans downward, these many smaller features grow and connect, eventually connecting to form larger cycles corresponding to the three glands of the original image. Fig. 3d shows this as a thresholded binarization of the red plane in Fig. 3c. As the plane pans through the level-set, components appear, grow, and connect. When they appear this corresponds to a birth. When they connect, the original component ceases to exist and a new component is formed; thus, they “die”. The persistence diagram for this example histology image is shown in Fig. 3e. The further off the diagonal a component is, the greater its persistence. Black triangles represent cycles (one-dimensional features). The blue band corresponds to features captured earlier in the filtration while the red band corresponds to features captured later in the filtration, where the components have merged to form three cycles, clearly matching the three cycles present as glands in the original image. Our hypothesis was that the size, number, and distribution of homology features would vary in accordance with the increasing neoplastic differentiation of PCa, so we sought to test this in real PCa histopathology images.

### Materials and Methods

#### Image Acquisition

We obtained 77 Hematoxylin and Eosin (H&E) stained histologic sections from radical prostatectomies derived from 19 patients at Tulane University Medical Center. Patients were selected to ensure a balanced representation of Gleason grades 3 through 5. Whole slide images from each patient case were selected based on maximum tumor area. Patients gave informed consent under a Tulane University Institutional Review Board (IRB) approved protocol. All methods were carried out in accordance with the approved guidelines and regulations. Specimens were digitized utilizing a digital whole slide scanner (Aperio CS2) at 20x magnification with 0.5 μm per pixel resolution. The resultant whole slide images (WSI’s) were scored manually by Dr. Andrew B. Sholl, a genitourinary pathologist at the Tulane University School of Medicine, with regions corresponding to purely graded tumor content outlined by hand in the Leica ImageScope software package (v. 12.2.2.5015). The corresponding annotations were then extracted manually and divided into 512 × 512 pixel regions of interest (ROIs) (corresponding to 256 × 256 μm^2^ regions). The process by which annotations and ROIs were extracted is shown in more detail in Fig. S1. A visual summary of the methods is shown in Fig. S2. A dataset of 5,182 ROI’s was generated from 77 digitized WSI’s, derived from 19 patients in total. A detailed breakdown of the ROIs, slides, and corresponding patients by grade is shown in Table 1. In addition, the histological characteristics of all participating patients are available in Table S1. Each ROI is a three-dimensional array of pixel intensities of dimension 512 × 512 × 3 comprised of three 512 × 512 arrays of pixel intensities of the red, green, and blue color channels of the RGB colorspace. Pixel intensities of each color channel correspond to integer values in the range 0 to 255.

**Table 1:** Image statistics by Gleason grade. A detailed breakdown of the number of patients, individual WSIs, and corresponding ROIs for each Gleason grade, from 3 to 5. Patient and slide counts are not necessarily exclusive, and may contain some overlap, i.e. in a Gleason 3 + 4 where both Gleason 3 and Gleason 4 are present.

Grade	Patient Count	Slide Count	ROI Count
Gleason 3	16	56	2,567
Gleason 4	16	45	2,351
Gleason 5	7	13	264

#### Color Normalization

To ensure the quality and reliability of downstream image analysis, color batch effects due to differences in stain preparation must be corrected. Color normalization was performed to minimize variance introduced from differences in H&E stain preparations, ensuring that artifacts from staining differences did not bias the ability of persistent homology to identify differences in tissue architecture. Color normalization was accomplished by the method originally described by Reinhard *et al*.^22^ and applied to H&E images by Wang *et al*.^23^. In accordance with this method the color characteristics of a source image (a Gleason 4 ROI with ideal staining properties) were transferred to a target image (each ROI). The source and target images were converted to the LAB colorspace and the mean and standard deviation (SD) of each axes of the LAB space were computed. The means of each of the target images LAB axes were subtracted from each pixel value in the target. The target image was then scaled by a factor determined from the SD of the source image. Then the mean of the source images LAB axes was added to the corresponding axes in the target image. Finally, the target image was converted back to the RGB colorspace.

#### Color Deconvolution

Hematoxylin imparts a blue-purple color and is specific to cell nuclei. Eosin imparts a pink color and binds to proteins non-specifically, resulting in broad staining of the cytoplasm and extracellular matrix. This produces essential contrast for the pathologist to identify key morphological features. Of interest to us are the spatial distribution and morphological characteristics of glands and individual PCa cells within ROIs. To capture these features of glands, only the nuclei are necessary, as a gland corresponds to an intraluminal space circumscribed by nuclei. Therefore, we need to extract the hematoxylin color channel from each ROI. As a result of overlapping absorption spectra, it is difficult to quantify the relative contribution of a stain for any given wavelength. Instead, the absorbance values of stain mixtures must be decomposed into absorbance values for single stains, a method known as color deconvolution^24^. Color deconvolution was performed in MATLAB^25^ using the Stain Normalization Toolbox developed by The University of Warwick in accordance with the process described by Macenko *et al*.^26^. This method transforms detected RBG intensities from the transmission of light through a stained specimen to a linear representation, defined as optical density (OD), where OD is linear with respect to the concentration of absorbing stain, facilitating the separation and quantification of individual stains. OD can then be computed for each of the RGB channels and the relative contribution of a single stain in each channel returned as a grayscale image. The hematoxylin color channel, corresponding to nuclei of glands and stroma, was extracted from each ROI as a 2D array of pixel intensities of dimension 512 × 512. Each pixel corresponds to an intensity value of hematoxylin staining in the interval [0,255]. The pixel values were then transformed by linear rescaling from the interval [0,255] to the interval [0,1] with 0 representing total black, and 1 pure white. This output image was used as the input for computing topological persistence.

#### Computing Persistence

Each ROI processed as described above, corresponding to a 2D array of scaled pixel intensities, can be considered as a function *f*: *I* → [0, 1] that maps the pixel at the coordinate (*x*, *y*) to a grayscale value *f*(*x*, *y*) in the interval [0, 1]^27,28^. A filtration over the sublevel set *L*_*τ*_ = {*x*: *f*(*x*) ≤ *τ*}, was computed using the R package TDA^29^. The output corresponds to two groups, the zero-dimensional homology group, *H*_0_(*L*_*τ*_), which is generated by connected components, and the one-dimensional homology group, *H*_1_(*L*_*τ*_), which is generated by cycles within the sublevel set.

#### Bootstrap Sampling

To ensure a balanced dataset, the 5,182 computed persistence diagrams were divided into three classes, Gleason 3, 4, and 5 as indicated by Table 1, and subsampled equally from each class for 100 bootstraps. The subsample size was 264 for each class, corresponding to the total number of Gleason 5 ROIs, which were less prevalent in the patient population with respect to Gleason 3 and 4. For each of 100 bootstraps, 264 Gleason 3 and Gleason 4 persistence diagrams were randomly sampled from their respective classes, yielding 100 balanced bootstrap samples comprised of 264 Gleason 3, Gleason 4, and Gleason 5 persistence diagrams, respectively. By restricting the subsample to the total number of Gleason 5 ROIs, a balanced dataset was generated, and by performing 100 bootstraps the stability of the subsampled results can be evaluated across bootstraps.

#### Ranked Persistence

The persistence features for each homology group were rank ordered in descending order by persistence (birth - death) into two vectors encompassing *H*_0_(*L*_*τ*_) (connected components) and *H*_1_(*L*_*τ*_) (cycles), respectively. The lengths of the *H*_0_(*L*_*τ*_) and *H*_1_(*L*_*τ*_) vectors were determined by finding the minimum count of *H*_0_(*l*_*τ*_) and *H*_1_(*L*_*τ*_) across all 792 ROI persistence diagrams for each bootstrap. The mean vector length across all bootstraps corresponded to a mean *H*_0_(*l*_*τ*_) length of 4,605.38 ± 800.89 and a mean *H*_1_(*L*_*τ*_) length of 4,044.49 ± 862.00. For each bootstrap, the *H*_0_(*L*_*τ*_) and *H*_1_(*L*_*τ*_) vectors were concatenated to form a single ranked persistence vector. The mean length of the ranked persistence vectors across bootstraps is 8,649.87 ± 1,642.21. See Table 2 for a summary of the ranked persistence vectors.

**Table 2:** Summary of ranked persistence features. Each vector corresponds to persistence for a given persistence diagram sorted in descending order such that **H**_*n* + 1_ ≤ **H**_*n*_ for all *n* ∈ *N*, where *N* is the minimum count of the *H*_*n*_ homological features and **H** is the vector comprising the homological features (*H*_*n*_). The mean vector length over all bootstraps, with standard deviation, is shown.

Feature	Mean Vector Length
Connected Components (*H*_0_(*l*_*τ*_))	4,605.38 ± 800.89
Cycles (*H*_1_(*l*_*τ*_))	4,044.49 ± 862.00

#### Dimensionality Reduction Using PCA

Principal component analysis (PCA) was performed for each ranked persistence vector across all 100 bootstraps. The first six principal components were selected from each bootstrap as they corresponded to at least 99% of the explained variance consistently across all bootstraps. The PCA factor loadings are shown in Fig. S3.

#### Hierarchical Ward Clustering

Hierarchical Ward clustering was selected as the optimal clustering method by evaluating multiple hierarchical clustering approaches and selecting the method that best delineated Gleason 3, 4 and 5 classes. The selection of hierarchical method was validated by performing agglomerative hierarchical clustering across bootstraps for Ward’s Method, Single-Linkage, Complete-Linkage, and Average-Linkage and computing the agglomerative coefficient (*AC*), a measure of the strength of the clustering approach, across all bootstraps. Ward’s method produced the highest *AC*, 0.992716 ± 0.000376. The optimal number of *k* clusters was determined by computing the Gap statistic for *k* from 2 to 10 across all bootstraps and selecting the maximum mean, corresponding to a *k* = 6.

#### Meta Clustering

We associate with each cluster, over all bootstraps, a three-dimensional vector of the number of Gleason 3, 4, and 5 ROIs contained in the cluster. The number of Gleason 3, 4, and 5 ROIs for each cluster were considered as x, y, and z coordinates respectively, and plotted in ${{\mathbb{R}}}^{3}$, as shown in Fig. S5. Hierarchical Ward Clustering was applied to the ${{\mathbb{R}}}^{3}$ coordinates. The optimal *k* was selected by computing the Gap statistic for *k* from 1 to 10 and selecting the minimum *k* such that *Gap*(*k*) ≥ *Gap*(*k* + 1) − *s*_*k* +1_, where ${s}_{k}=s{d}_{k}\sqrt{1+\frac{1}{B}}$ and *B* is the number of bootstraps, yielding *k* = 6 (see Fig. S4). Centroids were computed for each of the six identified clusters. A representative bootstrap sample was generated by computing the Euclidean distance between each cluster across all bootstraps to the computed centroids. The bootstrap with the minimum distance from all clusters to the identified centroids in ${{\mathbb{R}}}^{3}$ was selected for visualization. The meta-clusters were resampled with replacement for 1000 bootstraps and the Jaccard similarities were computed between the original clusters and the most similar clusters in the bootstrapped data in order to assess cluster stability.

#### Visualization Using t-SNE

In order to visualize the 6D projection in 2D space t-Distributed Stochastic Neighbor Embedding (t-SNE)^30^, a method of Stochastic Neighbor Embedding (SNE) was implemented for dimensionality reduction. t-SNE is well-suited to dimensionality reduction of high-dimensional data, as it retains local structure of the data from the higher-dimension in the lower-dimensional projection. SNE functions by converting high-dimensional Euclidean distances and their low-dimensional representation into conditional probabilities that correspond to similarities. SNE then minimizes the difference between a low-dimensional representation and the original high-dimensional representation using a gradient descent method. A perplexity, *p*, of 47 was chosen, corresponding to a smoothing measure of *k* nearest neighbors. t-SNE is robust to changes in *p*, with typically selected values ranging from 5 to 50^30^. The *p* of 47 was chosen by iterating *p* from one to 200 across all bootstraps, and computing the pseudo Bayesian Information Criteria (pBIC) at each *p*^31^. The pBIC provides an automatic way to estimate an optimal *p* by balancing KL-Divergence and *p*.

3 $$pBIC(p)=2KL(P||Q)+log(n)\frac{p}{n}$$

*p* was selected such that the pBIC for *p* from 5 to 50 over all bootstraps was minimized, as shown on Fig. S6. The resultant t-SNE projection corresponds to 792 points, 264 each of Gleason patterns 3, 4, and 5, projected into a 2D plane for each bootstrap sub-sample. The t-SNE result for the representative bootstrap sample with the minimum Euclidean distance to the compute meta-cluster centroid was plotted and colored according to the six clusters identified for that bootstrap using Ward hierarchical clustering.

#### Computing Averaged Persistence Intensity

Intensity diagrams were computed for the persistence diagram generated from each ROI in accordance with the method described by Chen, *et al*. in^32^. Persistence intensity diagrams are computed on persistence diagrams to facilitate non-parametric statistics. Intensity diagrams represent persistence diagrams as 2D probability density functions by placing a Gaussian kernel over every persistence point, weighted by distance from the diagonal (Death-Birth). This serves to place less weight on features with very short persistence. Given a diagram *D* comprised of persistence points (*b*_1_, *d*_1_), (*b*_2_, *d*_2_), …, (*b*_*N*_, *d*_*N*_), the persistence intensity is given by:

4 $${\hat{K}}_{\tau }(x,y)=\sum _{i\mathrm{=1}}^{N}({d}_{i}-{b}_{i})\frac{1}{{\tau }^{2}}K(\frac{x-{b}_{i}}{\tau })K(\frac{y-{d}_{i}}{\tau })$$

where *K* is a Gaussian kernel and *τ* is a smoothing parameter^32^. Intensity diagrams were computed for zero-dimensional and one-dimensional features independently. The smoothing parameter *τ* was set at 0.05 in order to introduce minimal smoothing while preserving individual persistence features. Computed zero-dimensional and one-dimensional intensity diagrams were then averaged by the clusters of the medoids of each of the six identified meta-clusters, to generate an averaged representation of intensity by medoids. The resulting averaged zero-dimensional and one-dimensional intensity diagrams were then plotted.

## Results and Discussion

### Persistent Homology and Unsupervised Clustering Reveals 6 Architectural PCa Subgroups across 3 Gleason Patterns

**Figure 4:**
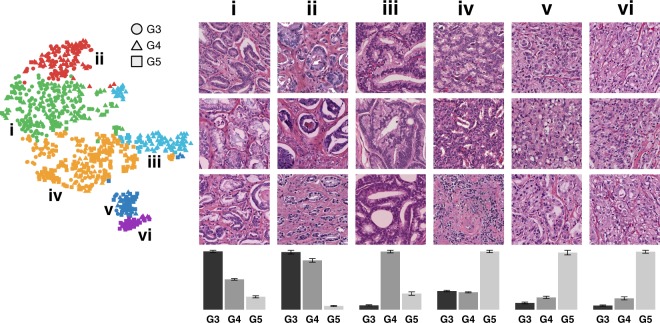
PH reveals a continuum of 6 architectural subpatterns across 3 Gleason patterns. t-SNE dimensionality reduction with corresponding Ward hierarchical clustering is shown for an example bootstrap, selected by finding the bootstrap with the minimal Euclidean distance between the six clusters of the bootstrap and the six medoid’s of the meta-clusters. Colors indicate unique clusters identified by Ward hierarchical clustering for the specific bootstrap represented. Circles, triangles, and squares correspond to purely graded ROI’s of Gleason 3, 4, and 5, respectively. Representative ROI’s from each cluster in the bootstrap are shown in numbered columns with each Roman numeral corresponding to a cluster with the same label. Each column is labeled with a histogram indicating the relative distribution of purely graded Gleason 3, 4, and 5 ROIs for each meta-cluster centroid, with error bars indicating standard error for the clusters within each meta-cluster.

After dimensionality reduction (PCA), clustering (Hierarchical Ward’s Method) of the ranked persistence vectors, and meta-clustering of the original clusters, six clusters emerged (visualized using t-SNE), shown in Fig. 4, ordered according to the most prevalent Gleason pattern as determined by the dominant proportion of purely graded ROIs in each meta-cluster. These six clusters, when arranged in order of increasing aggressiveness from Gleason pattern 3 to 5, demonstrate an architectural continuum transitioning from well differentiated to very poorly differentiated adenocarcinoma. A mean Jaccard coefficient of 0.7607 ± 0.1585 indicate that these six clusters are stable across bootstrap samples.

#### Overall Persistent Homology Continuum

**Figure 5:**
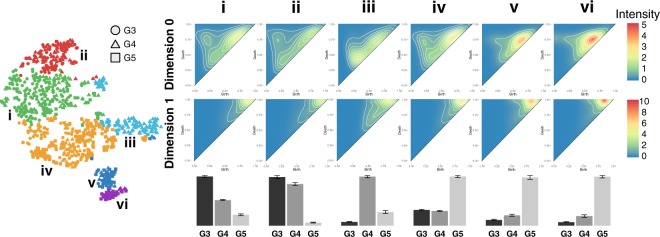
A continuum of homology features correspond to an architectural progression from Gleason 3 to Gleason 5. t-SNE dimensionality reduction with corresponding Ward hierarchical clustering is shown for an example bootstrap, selected by finding the bootstrap with the minimal Euclidean distance between the six clusters of the bootstrap and the six medoid’s of the meta-clusters. Circles, triangles, and squares correspond to purely graded ROI’s of Gleason 3, 4, and 5, respectively. Each column is labeled with a histogram indicating the relative distribution of purely graded Gleason 3, 4, and 5 ROIs for each meta-cluster centroid, with error bars indicating standard error for the clusters within each meta-cluster. Average intensity diagrams for the medoid of each meta-cluster are shown in numbered columns with each number corresponding to a cluster of the example bootstrap with the same label. The top row of intensity diagrams correspond to zero-dimensional homology features (connected components) and the bottom row of intensity diagrams correspond to one-dimensional homology features (cycles).

Column **i** corresponds to a predominantly Gleason 3 cluster dominated by a crowded glandular architectural pattern, as depicted in Fig. 2. Column **ii** corresponds to a predominantly Gleason 3 cluster dominated by a loosely-packed glandular pattern. Column **iii** corresponds to predominantly Gleason 4 cribriform pattern. Cluster **iv** corresponds to a predominantly Gleason 5 cluster dominated by a solid single-cell infiltrating architectural patterns. Column **v** and column **vi** correspond to predominantly Gleason 5 clusters dominated by single cell patterns with the presence of some poorly formed glands. The most compelling characteristic of this continuum is its division of dominant Gleason patterns into multiple clusters along the continuum. This indicates that there exist architectural subtypes within Gleason patterns, as well as subtypes that bridge Gleason patterns, and PH captures these differences. Persistence intensity diagrams map the differences in architecture between clusters to the underlying PH by providing an averaged representation of the persistence diagrams for each cluster, as shown in Fig. 5.

#### Zero-Dimensional Features and their Relation to PCa Architecture

By computing the persistence intensity diagrams for the zero-dimensional homology features (connected components) and one-dimensional homology features (cycles) independently we are able to evaluate the contribution of each feature to the delineation of clusters by architecture. Zero-dimensional components comprise connected components, and their presence and distribution can serve as a representation of nuclear density. In Fig. 3d this is demonstrated in the top threshold, representing a particular threshold of the filtration through the sub-level set, which is insufficient to capture the example glands as one-dimensional cycles. Instead, as glands are comprised of nuclei that are not evenly distributed around the perimeter of the gland, the gland is represented by a series of zero-dimensional connected components that have failed to merge into cycles. Thus, the density and distribution of nuclei controls when connected components merge into cycles with sparsely populated nuclei merging later in the filtration or with densely populated nuclei merging earlier in the filtration. A trifurcation emerged in the zero-dimensional connected components, as evidenced by the upper row of intensity diagrams of Fig. 5. Three clear classes of connected components are shown: The lower left of the zero-dimensional intensity diagram corresponds to early birth, early death features (connected components that appear early and persist for a short span). Early birth/early death features (small off-diagonal distance) may be considered as noise in one-dimensional features, but in zero-dimensional features they provide insight into the density of nuclei in a given ROI, with more connected components appearing for more densely populated nuclei. The upper left of the zero-dimensional intensity diagram corresponds to early birth and long persistence (connected components that appear early, but persist for a long span). These early-birth/late-death features correspond to larger connected components, most likely glands and clusters of glands that persist through the filtration. Finally, the upper right of the zero-dimensional intensity diagram corresponds to late birth and short persistence (connected components that appear much later, but persist a short span). These late-birth features correspond to connected components that do not originate until later in the filtration, indicating that they arise from more sparsely populated regions, likely stroma. Differences in the distribution of these three zero-dimensional sub-classes are linked to clear architectural difference in the ROI’s.

#### One-Dimensional Features and their Relation to PCa Architecture

Likewise, the distribution of one-dimensional cycles, for which a subset of the filtration corresponds to glands, correspond to architectural differences captured in the ROIs. One-dimensional intensity diagrams, as shown in the bottom row of Fig. 5 have a single prominent cluster, as opposed to the three identified in the zero-dimensional diagrams. This cluster varies both in the width of distribution along the diagonal (corresponding to birth time) and the distribution of diagonal distance (corresponding to persistence).

Early birth/short persistence corresponds to features that are densely populated and captured early in the sublevel set filtration, so connect early, but are destroyed as they merge into other connected components. The Gleason 5 solid pattern shown in Fig. 2, and captured by cluster **iv**, exhibits this pattern of tightly packed sheets of nuclei with minimal intervening stroma. This pattern is shown prevalently in cluster **iv** in Fig. 4, corresponding to the top intensity diagram shown in cluster **iv** in Fig. 5. This cluster is dominated by a significant proportion of solid Gleason 5 pattern ROI’s, and this is reflected in the high concentration of connected components that are birthed early, and persist a short time until they merge into larger connected components.

The lower row of Fig. 5, corresponding to one-dimensional cycles, shows a clear progression from Gleason 3 to Gleason 5 pattern clusters, with an overall decrease in the width of the distribution of birth times along the diagonal. The width of this distribution corresponds to the variance in nuclei density of birthed one-dimensional cycles. Gleason 3 ROIs are comprised of well circumscribed glands consisting of a ring of epithelial nuclei, and surrounded by intervening stroma with sparsely populated stromal nuclei. This results in a wider distribution of birthed cycles as features such as glands, comprised of densely arranged nuclei, are birthed early, and stromal nuclei that connect into loop like structures, due to their sparsity, are birthed late in the filtration. As the adenocarcinoma becomes less differentiated moving from Gleason 3 to Gleason 5 patterns, as evidenced by Fig. 4, the width of the birth time distribution narrows, until it reaches its smallest diameter in cluster **vi** in Fig. 5, which corresponds to single-cell pattern Gleason 5 characterized by a total loss of differentiation as cancer cells invade the surrounding stroma.

### Persistent Homology Groups within Gleason Pattern 3

**Figure 6:**
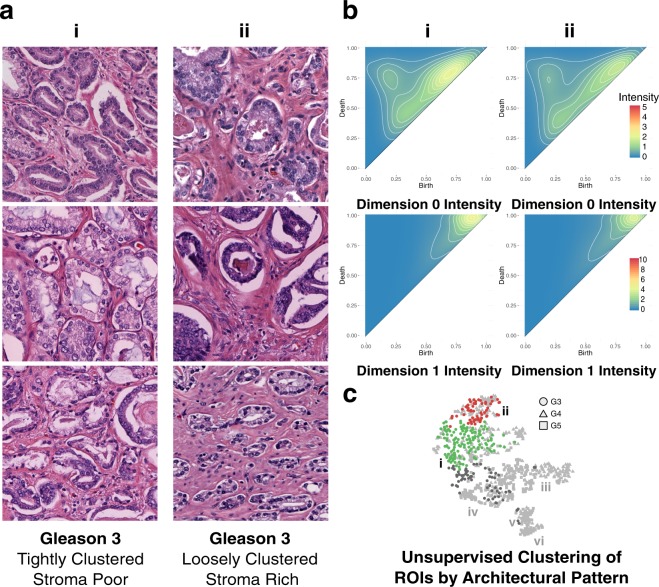
Persistent homology differentiates Gleason 3 loosely clustered, stroma rich pattern from tightly clustered, stroma poor pattern. (**a**) Representative Gleason 3 ROIs from green cluster i, comprising tightly clustered, stroma poor pattern ROIs, and red cluster ii, comprising loosely clustered, stroma rich pattern ROIs. (**b**) Zero-dimensional and one-dimensional persistence intensity diagrams, for cluster i and ii respectively, with intensity values ranging 0 to 5 for zero-dimensional diagrams, and 0 to 10 for one-dimensional diagrams, as indicated by the included legend. (**c**) Hierarchical Ward clustering results of first six principle components, with t-SNE dimensionality reduction for visualization, with unique clusters indicated by color, and Gleason pattern indicated by shape (Gleason 3: circle; Gleason 4: triangle; Gleason 5: square). Relevant clusters i (green) and ii (red) are labeled accordingly. Light grey points correspond to Gleason 4 and Gleason 5 points present in all clusters, but not considered in this figure. Dark grey points correspond to Gleason 3 points present in clusters other than the two being considered in this figure.

Shown in Fig. 6 is a comparison of the two clusters, from the representative bootstrap, with the highest proportion of Gleason 3 ROIs, cluster **i** and cluster **ii**. Cluster **i** is comprised of 58.58% Gleason 3, 28.87% Gleason 4 and 12.55% Gleason 5 pattern ROIs. The dominant architectural pattern is tightly clustered Gleason 3 pattern ROIs with a minimal amount of intervening stroma, shown in Fig. 6a. Cluster **ii** is comprised of 47.11% Gleason 3, 51.24% Gleason 4 and 0.02% Gleason 5 pattern ROIs. The dominant architectural pattern corresponds to loosely clustered Gleason 3 pattern ROIs with significant intervening stroma. These differences are captured in the persistence intensity diagrams shown in Fig. 6b. Moving from the zero-dimensional connected component intensity diagram of cluster **ii** to that of cluster **i** shows merging of the three dominant zero-dimensional features, corresponding to an increase in the homogeneity of the distribution of glands as the intervening stroma is lost between cluster **ii** and **i**. The one-dimensional cycle intensity diagrams for clusters **ii** and **i** also exhibit a narrowing of the distribution of persistent cycles moving from cluster **ii** to **i**. This narrowing is indicated by the shift of intensity from cluster **ii** up the diagonal in cluster **i**. This behavior is consistent with the architectural differences between clusters; as the stroma (and corresponding stromal nuclei) are lost in cluster **i**, with respect to cluster **ii**, the scale of persistent cycles will narrow.

### Persistent Homology Groups within Gleason Pattern 4

**Figure 7:**
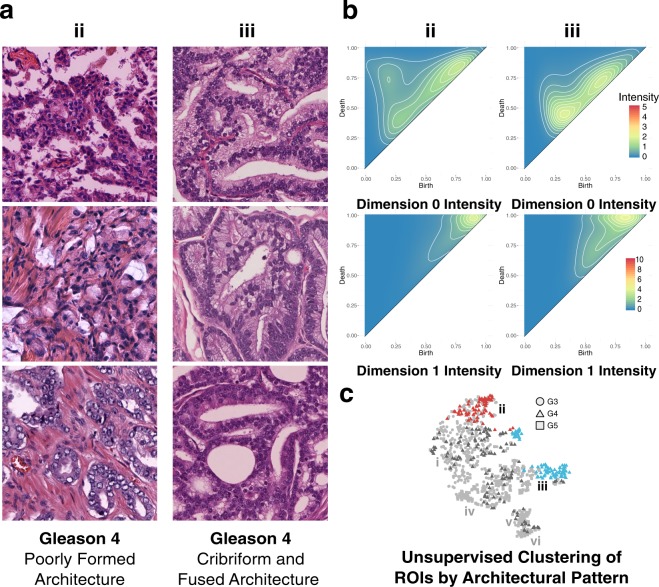
Persistent homology differentiates Gleason 4 poorly formed pattern from cribriform and fused pattern. (**a**) Representative Gleason 4 ROIs from green cluster **iii**, comprising cribriform and corded pattern ROIs with attenuated lumen, and blue cluster **iv**, comprising fused pattern ROIs with lumen preservation. (**b**) Zero-dimensional and one-dimensional persistence intensity diagrams, for cluster **ii** and **iii** respectively, with intensity values ranging 0 to 5 for zero-dimensional diagrams, and 0 to 10 for one-dimensional diagrams, as indicated by the included legend. (**c**) Hierarchical Ward clustering results of first six principle components, with t-SNE dimensionality reduction for visualization, with unique clusters indicated by color, and Gleason pattern indicated by shape (Gleason 3: circle; Gleason 4: triangle; Gleason 5: square). Relevant clusters **ii** (red) and **iii** (blue) are labeled accordingly. Light grey points correspond to Gleason 3 and Gleason 5 points present in all clusters, but not considered in this figure. Dark grey points correspond to Gleason 4 points present in clusters other than the two being considered in this figure.

Shown in Fig. 7 is a comparison of the two clusters, from the representative bootstrap, with the highest proportion of Gleason 4 ROIs, cluster **ii** and cluster **iii**. Cluster **ii** is comprised of 51.24% Gleason 4, 47.11% Gleason 3, and 0.02% Gleason 5 pattern ROIs. The dominant architectural patterns present within cluster **ii** correspond to poorly formed glands, as shown in Fig. 7a. Cluster **iii** is comprised of 80.41% Gleason 4, 0.02% Gleason 3, and 17.53% Gleason 5 pattern ROIs. The dominant architectural patterns present in cluster **iii** are almost exclusively cribriform, as shown in Fig. 7a. Cluster **iii**, due to the presence of a cribriform architecture and the loss of well defined lumens, is characterized as being more aggressive. This increase in aggressiveness, associated with the presence of a cribriform architecture, is consistent with Fig. 7c, which shows cluster **iii** clustering closest to cluster **iv**, a cluster dominated by Gleason 5 patterns, indicating that the architecture of cluster **iii** is more similar architecturally to aggressive architectural subtypes than that of cluster **ii**. The topological differences between clusters are captured in the persistence intensity diagrams shown in Fig. 7b. Moving from the zero-dimensional connected component intensity diagram of cluster **ii** to that of cluster **iii** shows merging of the three dominant zero-dimensional feature groups into two dominant groups, corresponding to an increase in the homogeneity of architecture between the one-dimensional cycle intensity diagrams between clusters. Clusters **ii** and **iii** also exhibit a widening of the distribution of persistent cycles moving from cluster **ii** to **iii**. This widening is indicated by the shift of intensity from cluster **ii** downwards along the diagonal in cluster **iii**. This behavior is consistent with the architectural differences between clusters; cluster **ii**, as a result of the dominant poorly formed gland architecture, exhibits rudimentary lumen formation, and thus fewer cycles in both number and scale. Cluster **iii**’s dominant cribriform pattern is characterized by well-formed lumens, and as such exhibits a wider range of cycles, as indicated by the increase in both the width of the distribution of persistent cycles in Fig. 7b-**iii**’s intensity diagram, as well as the height (distance off-axis) indicative of the presence of larger cycles (the well formed cribriform lumen).

Our results, which may indicate that the cribriform architecture is topologically similar to known aggressive PCa architectures, are supported by recent work that has shown that patients with cribriform pattern Gleason 4 subtypes are at a higher risk for treatment failure than those with other architectural subtypes^20^. In addition, the presence of cribriform patterns in patients having undergone radical prostatectomy is associated with an increased likelihood of biochemical recurrence, extraprostatic extension, positive surgical margins, and the presence of distant metastases^19,33–37^. The characteristics associated with cribriform pattern are consistent with the patient specific histology of this study as only two of the nineteen patients included in this study exhibited regional lymph node metastases, and both were present in cluster **iii**, corresponding to 59.79% of the ROIs present in the cluster. Additionally, both patients exhibited margin involvement, (present in only 36.84% of patients), extraprostatic extension (present in 52.63% of patients), and seminal vesicle invasion (present in only 15.79% of patients and for which the only two incidences of bilateral invasion were confined to these two patients). In addition, one of the two patients exhibited lymphovascular invasion (present in only 10.53% of patients.)

### Persistent Homology Groups within Gleason Pattern 5

**Figure 8:**
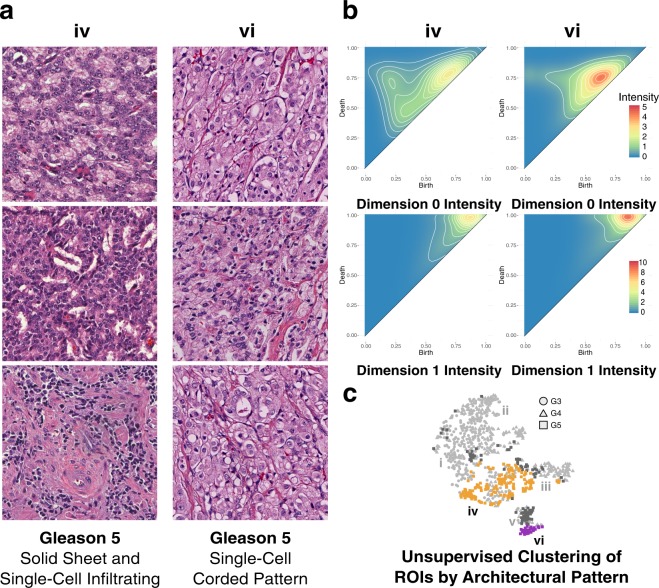
Persistent homology differentiates Gleason 5 solid sheet and single cell infiltrating pattern from single cell corded pattern. (**a**) Representative Gleason 5 ROIs from orange cluster iv comprising solid sheet pattern ROIs, and purple cluster vi comprising single cell pattern ROIs. (**b**) Zero-dimensional and one-dimensional persistence intensity diagrams, for cluster iv and vi respectively, with intensity values ranging 0 to 5 for zero-dimensional diagrams, and 0 to 10 for one-dimensional diagrams, as indicated by the included legend. (**c**) Hierarchical Ward clustering results of first six principle components, with t-SNE dimensionality reduction for visualization, with unique clusters indicated by color, and Gleason pattern indicated by shape (Gleason 3: circle; Gleason 4: triangle; Gleason 5: square). Relevant clusters iv (orange) and vi (purple) are labeled accordingly. Light grey points correspond to Gleason 3 and Gleason 4 points present in all clusters, but not considered in this figure. Dark grey points correspond to Gleason 5 points present in clusters other than the two being considered in this figure.

Shown in Fig. 8 is a comparison of two of the clusters, from the representative bootstrap, with the highest proportion of Gleason 5 ROIs, cluster **iv** and cluster **vi**. Cluster **v** is not shown for comparison as it is almost indistinguishable from cluster **vi** in both architecture (except for the absence of comedonecrosis) and persistence, as indicated by the zero and one-dimensional intensity diagrams. A more comprehensive breakdown of each cluster containing Gleason 5 ROIs, with corresponding representative images and intensity diagrams, is available in Fig. S9. Cluster **iv** is comprised of 61.13% Gleason 5, 24.70% Gleason 3, and 14.17% Gleason 4 pattern ROIs. The dominant architectural patterns present in cluster **iv** are solid sheet and single cell infiltrating with intermittent cords and poorly formed glands, as shown in Fig. 8a. Cluster **vi** is comprised of 77.14% Gleason 5, 22.86% Gleason 4 pattern ROIs, with no Gleason 3 patterns present. The dominant architectural pattern in cluster **vi** is almost exclusively single cell infiltrating with some rudimentary lumen formation, shown in Fig. 8a, characterized by single cancer cells visible in the stroma of the ROI, although 11.11% of the Gleason 5 pattern present is comedonecrosis. The comedonecrosis architecture is confined entirely to cluster **vi** and is present in no other clusters. This clustering of ROIs with comedonecrosis architecture present is consistent with the current Gleason grading consensus which requires upstaging of cribriform architectures with comedonecrosis present from Gleason 4 to Gleason 5^15,19,36,37^. The presence of a high concentration of late-birth zero-dimensional components in the single-cell pattern that dominates the architecture of cluster **vi** is expected as the single-cell pattern is comprised of uniformly distributed single cancer cells surrounded by intervening stroma. These zero-dimensional features persist for a significant interval as a consequence of the intervening stroma, before merging with other components, thus resulting in the birth of larger connected components later in the filtration. Moving from the zero-dimensional connected component intensity diagram of cluster **iv** to that of cluster **vi** shows a merging of the three dominant zero-dimensional feature groups into one, corresponding to an increase in the homogeneity of the distribution of cells as well as an increase in intervening stroma. The one-dimensional cycle intensity diagrams for cluster **iv** and **vi** also exhibit a narrowing of the distribution of persistent cycles moving from cluster **iv** to **vi**. This narrowing is indicated by the shift of intensity from cluster **iv** upwards along the diagonal in cluster **vi**.

### Gleason Grade Architectural Changes Across Persistent Homology Groups

**Figure 9:**
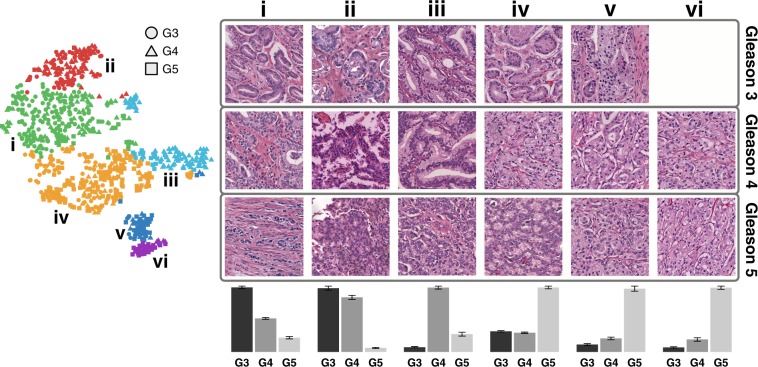
Gleason grade progression across clusters. Example ROIs for each putative Gleason grade 3, 4 and 5 are shown across clusters **i** to **vi**, when present. Columns correspond to clusters **i** to **vi** and rows correspond individual Gleason grades. Each column is labeled with a histogram indicating the relative distribution of purely graded Gleason 3, 4, and 5 ROIs for each meta-cluster centroid, with error bars indicating standard error for the clusters within each meta-cluster. To the left are hierarchical Ward clustering results of the first six principle components, with t-SNE dimensionality reduction for visualization, with unique clusters indicated by color, and Gleason pattern indicated by shape (Gleason 3: circle; Gleason 4: triangle; Gleason 5: square).

We have shown that that PH captures architectural differences between Gleason grades, as well as demonstrating differences in the same Gleason grade between two groups dominated by that grade. However, it is apparent that even within clusters that are dominated by a single Gleason grade, there exists a significant proportion of ROIs belonging to other Gleason grades (for example, Gleason grade 3 ROIs that are present within architectural clusters that are dominated by Gleason grade 5). To further investigate this we looked at ROIs within a particular Gleason grade across the continuum of architectural subgroups identified by PH and unsupervised clustering. Fig. 9 shows a summary of example ROIs for each Gleason grade, 3 to 5, for each cluster identified. This intriguing result indicates that Gleason grade ROIs that were placed in a cluster dominated by a different grade share architectural commonalities with that grade. For further details regarding the architectures of Gleason grades 3, 4, and 5 across clusters see Figs. S7, S8, and S9 respectively. The result confirms that PH is sensitive to architectural differences that distinguish PCa from ‘Gleason 3-like’ to ‘Gleason 5-like’. There are a couple of possible inferences we can draw from this result. The first is that, due to the limitations of the labeling approach used in this work, large areas of tissue labeled as a single putative Gleason grade by the pathologist rater actually contained within it smaller patches of PCa of a different grade, and that it is these labeling ‘errors’ that are being identified by PH when the small ROIs are taken out of context with the surrounding tissue. This could be avoided in the future by increasing the granularity of the labeling approach to annotate and label smaller areas in the whole slide images, although this would come at significant expense in terms of labor and time. Another possible inference that can be drawn is that ROIs from a low Gleason grade that are clustered with a higher Gleason grade are correctly labeled at the low Gleason grade, but in fact contain previously unappreciated architectural features that share more in common with higher Gleason grade architecture. An interesting study to investigate this would be to study the correlation between such cases, and patient outcomes, to determine whether PH is sensitive to architectural changes within a low Gleason grade that would have prognostic significance. This will be the subject of future work.

## Conclusions

In this paper, persistent homology is proposed as a new means of describing PCa architecture. Using persistent homology a continuum of architectural subtypes beyond Gleason grades is identified, consisting of six groups that each share common architectural features. These architectural features are captured through representations given by persistent homology as the scale and distribution of zero and one-dimensional persistence features. Persistent homology is a powerful tool for differentiating PCa, both by Gleason pattern and by morphological subpatterns within each Gleason pattern. The results of this study display the ability to stratify PCa into architectural subtypes using persistent homology as a tool, which ultimately may have different prognostic outcomes. Of particular interest is the ability to segregate PCa architecture into potentially prognostically distinct groups, such as Gleason 4 cribriform pattern. Cribriform Gleason pattern 4 has been shown to have poor prognostic outcomes when compared to other Gleason 4 patterns, and this finding was consistent with the more aggressive characteristics of the patient histology present within that PH  group. Similarly, the inclusion of comedonecrosis in the Gleason 5 pattern group serves to demonstrate that architectural differences may underlie prognostic differences. The sensitivity and robustness of PH to PCa architecture was further reinforced when studying the architectural features within a single Gleason grade across the unique architectural subgroups identified in this work. While this is a preliminary work and yields no definitive conclusions regarding clinical utility, it does point to the power of persistent homology to interrogate the sub-architectures present in PCa. Using this tool it may be possible to develop a better understanding of not only architectural subtypes in PCa but, more importantly, how they correlate with patient prognostic outcomes. In particular, this study demonstrates that PCa architecture is a continuum and addressing it as such may yield finer granularity in grading patient PCa, with the potential to guide more personalized prognostic decisions.

## Future Work

To most effectively leverage persistent homology for the discovery and classification of PCa architectures, it is necessary to have a robust understanding of persistent homology, ideal representations of persistence diagrams, and how the persistence of zero and one-dimensional features relate back to the architecture of PCa. What is noise and what has meaning in the context of persistent homology is highly domain specific, and requires extensive investigation. In addition, the use of clustering as an exploratory approach is highly dependent on the clustering method used, as well as the dimensionality reduction approach applied. Future research involves a larger clinical study with significantly larger patient recruitment, in order to build a larger database of purely graded ROIs to further interrogate differences both between and within Gleason patterns. Ultimately, the results of such a study will be used to generate quantitative descriptors of the morphology of prostate adenocarcinoma ROIs, which may then be applied to whole-slide images to generate an automated, quantitative heatmap of cancer aggressiveness. By incorporating persistent homology into a longitudinal study of patient outcomes, it may be possible to stratify PCa into Gleason sub-patterns that could then be correlated with patient prognostic outcomes. Ongoing work is aimed at investigating and optimizing approaches for the generation of an image library based on these descriptors and evaluating it against standard Gleason grade classifications, both architecturally, and prognostically.

## Supplementary information

Supplementary File

## Data Availability

De-identified H&E images are available for use in accordance with provision of informed consent under Tulane University Biomedical IRB project #623770-3. To obtain data contact J.Q.B.
