# Supplementary material for: Persistent Homology for the Quantitative Evaluation of Architectural Features in Prostate Cancer Histology
Source: Sci Rep. 2019 Feb 4;9:1139. doi: 10.1038/s41598-018-36798-y (PMC6361896; doi:10.1038/s41598-018-36798-y)
Supplement: Supplementary file 1 — Supplementary File [file 41598_2018_36798_MOESM1_ESM.pdf]

Supplementary Information

Persistent Homology for the Quantitative Evaluation of Architectural Features in Prostate Cancer Histology

Peter Lawson[1], Andrew Sholl[2], J. Quincy Brown[1,+], Brittany Terese Fasy[4,+], and Carola Wenk[3,+]

[1]Department of Biomedical Engineering, Tulane University, New Orleans, Louisiana, 70118, USA
[2]School of Medicine, Tulane University, New Orleans, Louisiana, 70118, USA
[3]Department of Computer Science, Tulane University, New Orleans, Louisiana, 70118, USA
[4]School of Computing and Department of Mathematical Sciences, Montana State University, Bozeman, Montana, 59717, USA
[+]These authors contributed equally to this work. Correspondence and requests for materials should be addressed to J.Q.B. (email: jqbrown@tulane.edu)

Table 1. Histological characteristics of patient cases. Histological characteristics extracted from the pathology reports of patient cases. Reported characteristics include the Gleason grade by case, AJCC staging of the case, the presence and location, if noted, of margin involvement, the presence of lymphovasculature or perineural invasion, and the presence, if noted, of additional non-tumor histological features. HG-PIN indicates high grade prostatic intraepithelial neoplasia.

Patient	Grade	AJCC Staging	Margin Involvement	Lymphovasculature Inv.	Perineural Inv.	Additional Non-Tumor
1	3+3, tertiary 4	pT2c, N0, Mx	Involved: Peripheral	Not Identified	Present	HG PIN, Inflammation: (Acute and Chronic), Nodular Prostatic Hyperplasia
2	3+4	pT3a, N0, Mx	Uninvolved	Not Identified	Present	HG PIN, Chronic Inflammation, Periglandular Venous Thrombosis
3	3+4	pT2c, N0, Mx	Involved: Right Posterior	Not Identified	Present	None Documented
4	3+4	pT2c, N0, Mx	Uninvolved	Not Identified	Not Identified	Inflammation: Chronic and Nodular Prostatic Hyperplasia
5	3+4	pT3a, N0, Mx	Uninvolved	Not Identified	Present	HG PIN, Inflamation (Chronic and Acute), Nodular Prostatic Hyperplasia, Cystic Atrophy
6	3+4	pT2c, N0, Mx	Uninvolved	Not Identified	Present	HG PIN, Inflamation (Acute and Chronic), Nodular Prostatic Hyperplasia
7	3+4	pT3a, N0, Mx	Uninvolved	Present	Present	HG PIN, Acute and Chronic Prostatitis with Granulomatous inflammation
8	3+4, tertiary 5	pT3a, N0, Mx	Uninvolved	Not Identified	Present	HG PIN, Chronic Inflammation, Nodular Prostatic Hyperplasia
9	3+4, tertiary 5	pT3a, N0, Mx	Involved	Not Identified	Present	HG PIN, Acute Supparative and Chronic Prostatitits, Nodular Prostatic Hyperplasia, Transitional Metaplasia
10	3+4, tertiary 5	pT2c, N0, Mx	Uninvolved	Not Identified	Present	HG PIN, Inflammation (Chronic)
11	3+5	pT2c, N0, Mx	Uninvolved	Not Identified	Present	HG PIN
12	4+3	pT2c, N0, Mx	Uninvolved	Not Identified	Present	HG PIN, Inflammation (Chronic), Nodular Prostatic Hyperplasia
13	4+3	pT3a, N0, Mx	Uninvolved	Not Identified	Present	HG PIN, Benign Prostatic Hyperplasia
14	4+3, tertiary 5	pT3b, N0, Mx	Benign	Not Identified	Present	HG PIN, Nodular Prostatic Hyperplasia, Inflammation (Chronic)
15	4+3, tertiary 5	pT2c, N0, Mx	Uninvolved	Not Identified	Present	Intermediate PIN, Inflammation (Acute and Chronic)
16	4+3, tertiary 5	pT2c, N0, Mx	Uninvolved	Not Identified	Present	HG PIN, Basal Cell Hyperplasia
17	4+3, tertiary 5	pT3b, N1, Mx	Involved: Peripheral Margin	Present	Present	Nodular Prostatic Hyperplasia, HG PIN, Prostatitis (Acute and Chronic) , Periprostatic Phlebolith
18	4+3, tertiary 5	pT3a, N0, Mx	Involved	Not Identified	Present	Inflammation, Nodular Prostatic Hyperplasia
19	5+4, tertiary 3	pT3b, N1, Mx	Involved: Right Posterior	Not Identified	Present	Prostatitis (Acute and Chronic)

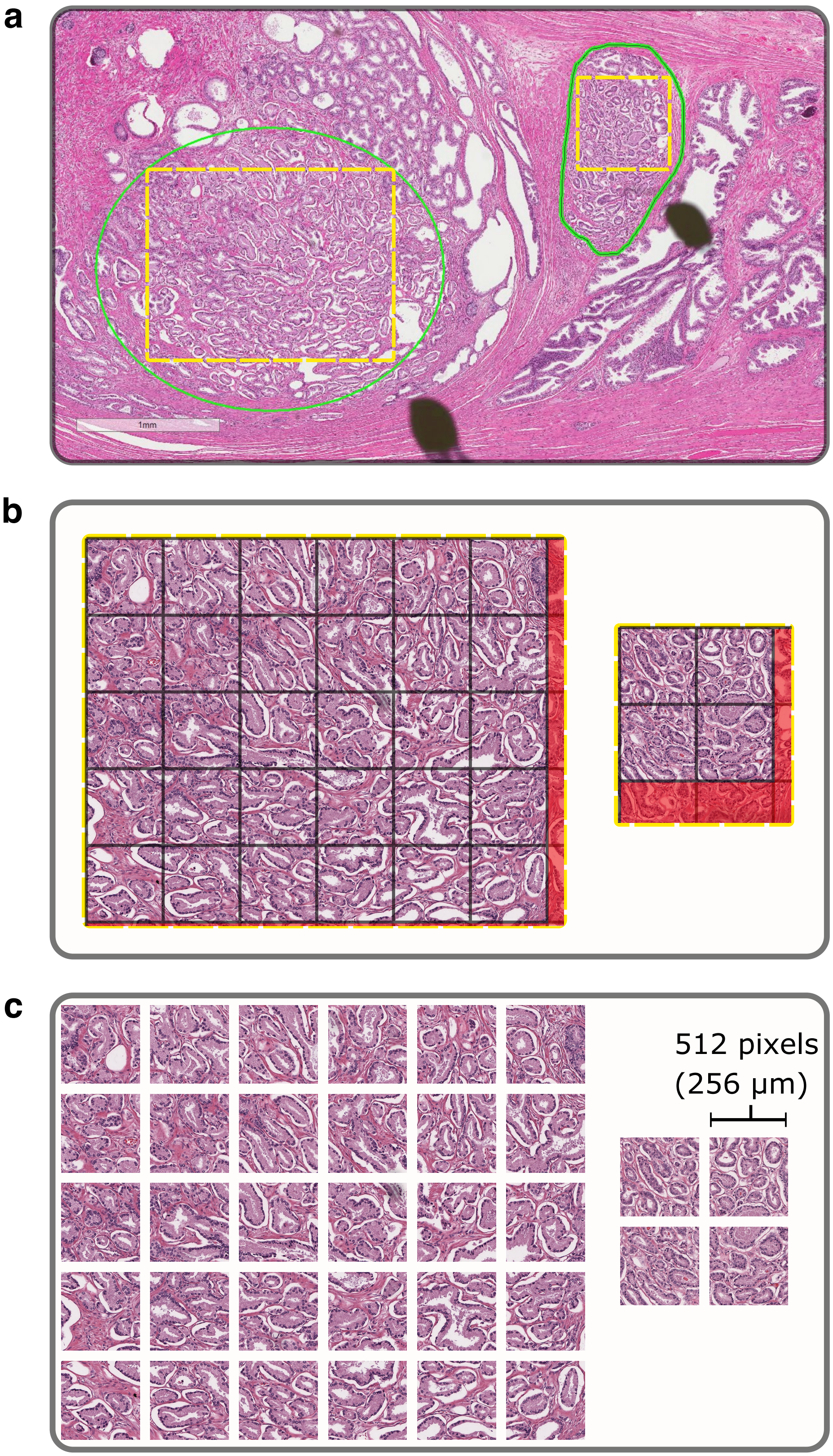

Figure 1. Process of acquiring annotated ROIs. a) Purely graded regions of Gleason 3, 4, and 5 are manually circumscribed (shown in green) by an expert genitourinary pathologist. A rectangle (in yellow) is extracted from each purely graded region manually. **b)** The extracted regions are automatically segmented into 512x512 pixel regions (indicated by the grid overlay). Regions that do not match this dimensional requirement are automatically removed (removed regions shown in red). **c)** The resultant ROIs are shown.

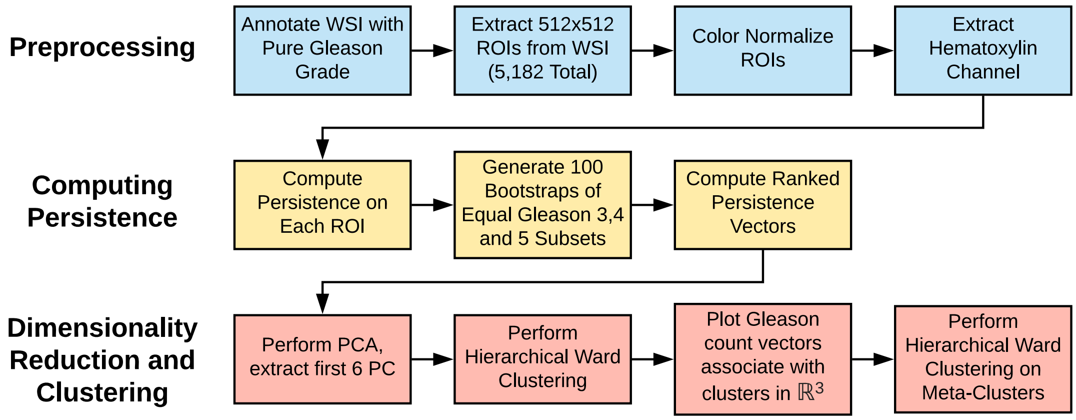

Figure 2. Summary of methodology. Represented in three stages; preprocessing (in blue), computing persistence (in yellow), and dimensionality reduction and clustering (in red).

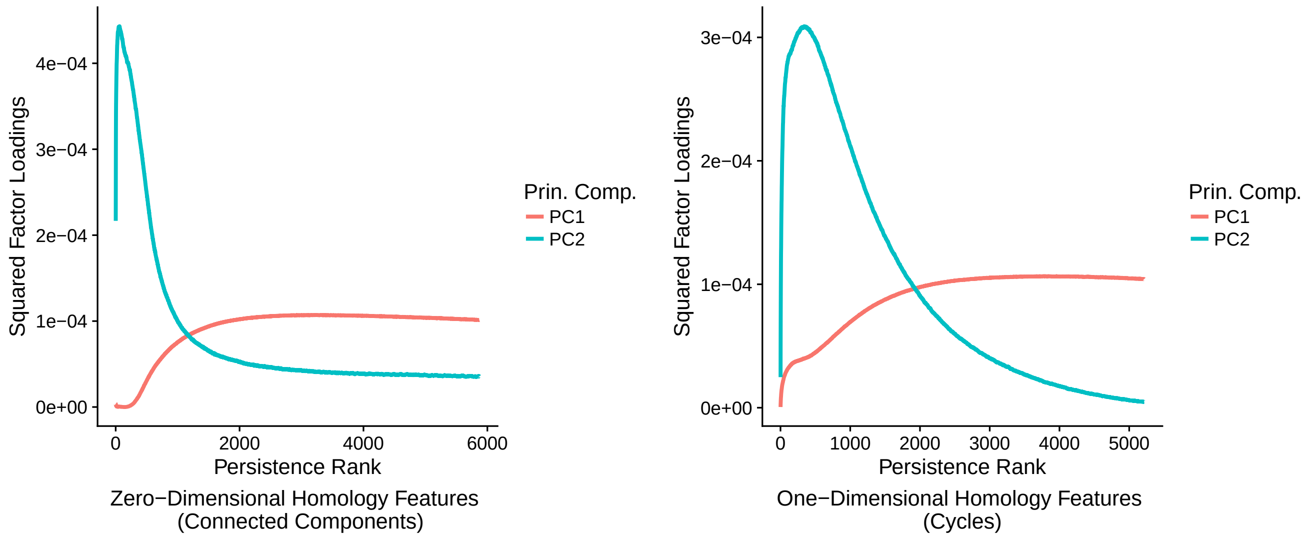

Figure 3. PCA Factor Loadings for the zero and one dimensional homology features. Factor loadings are shown for principle component one (77.72% of explained variance) and principle component two (14.04% of explained variance). The left plot corresponds to the zero dimensional homology features and the right plot corresponds to the one dimensional homology features. Squared factor loadings are shown, indicating the percentage of the variance explained by a factor.

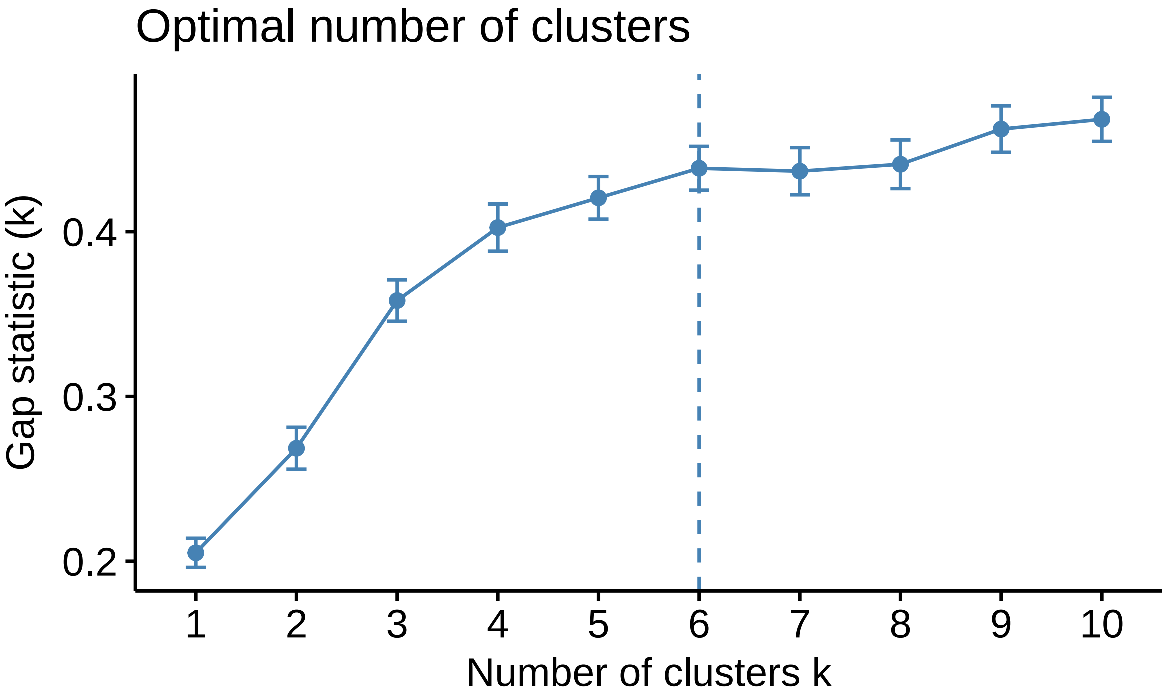

Figure 4. Selection of optimal *k* clusters for meta clustering. Gap statistic computed for $\mathbb{R}^3$ clusters using Ward hierarchical clustering ranging from *k* of 1 to 10, repeated over 100 bootstrap permutations. Error bars represent standard error. The optimal *k* is indicated with a blue vertical line.

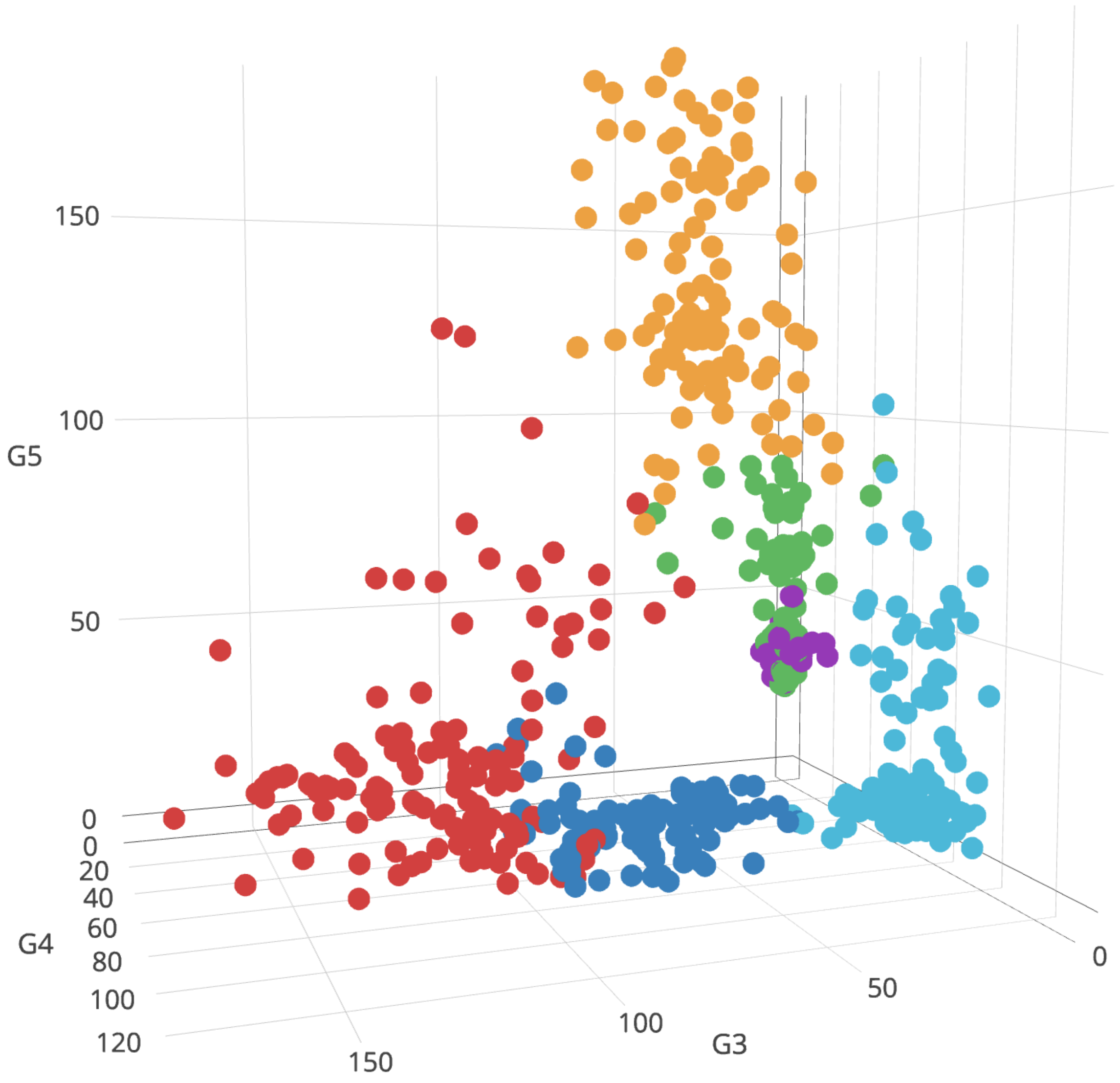

Figure 5. Visualization of meta clustering with identified clusters. 600 identified clusters (6 clusters per bootstrap, for 100 bootstraps) are shown in $\mathbb{R}^3$ by mapping Gleason 3, 4, and 5 frequencies per cluster to *xyz* coordinates. Hierarchical Ward Cluster results for a *k* of 6 are indicated with color.

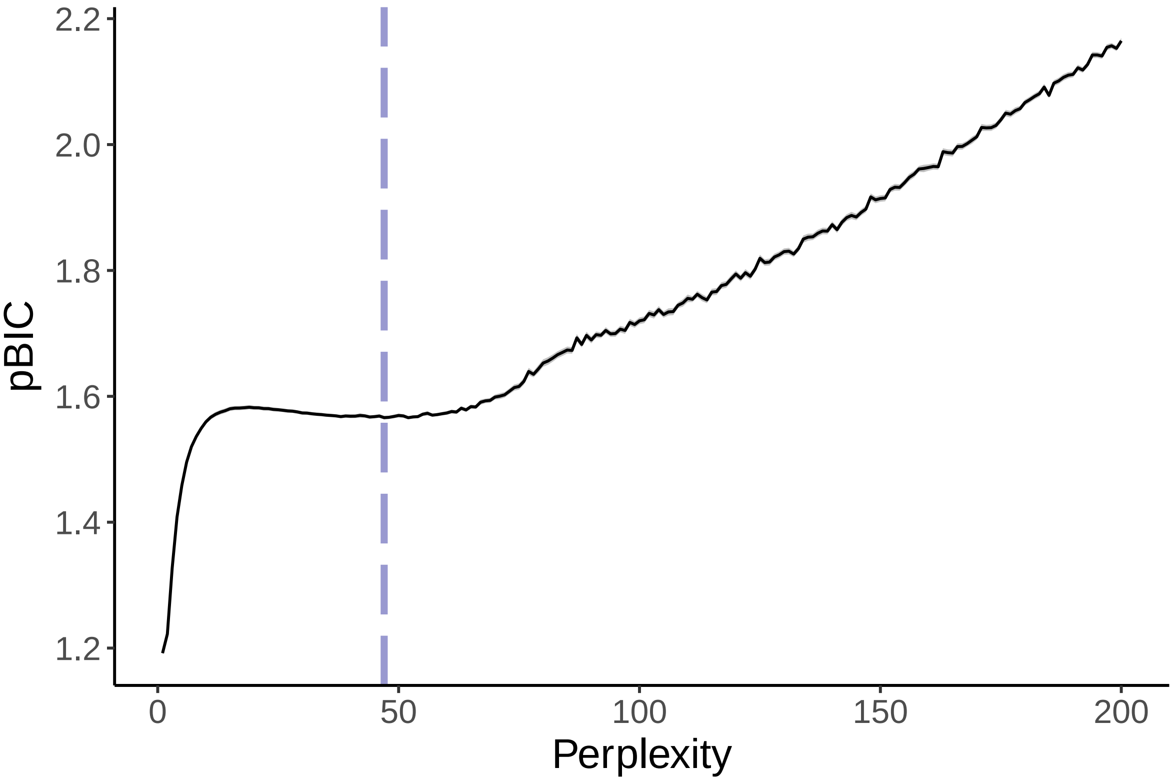

Figure 6. **Selection of optimal t-SNE perplexity.** pBIC computed for t-SNE embeddings ranging from perplexity of 1 to 200, repeated over 100 bootstrap resamplings of Gleason 3, 4, and 5 ROIs. Standard error of pBIC is shown in grey. The optimal t-SNE perplexity is indicated with a blue vertical line.

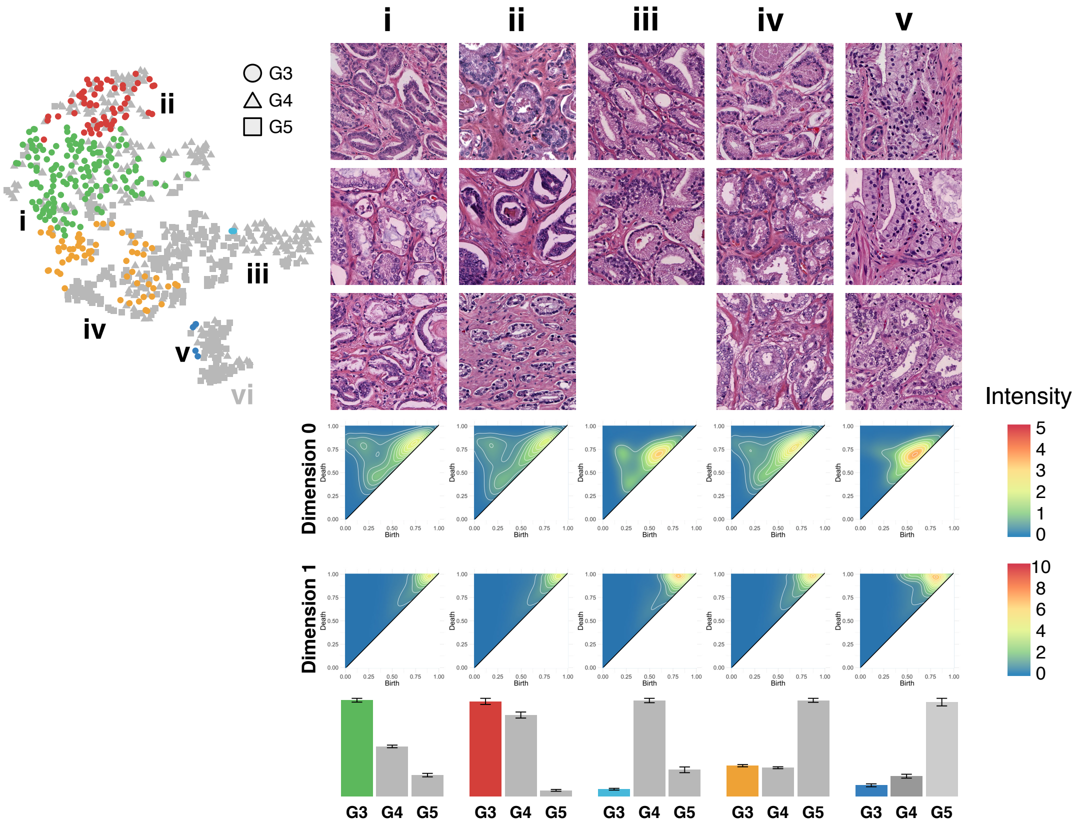

Figure 7. Comparison of Gleason 3 ROIs and intensity diagrams across all clusters. Example ROIs and averaged intensity diagrams for Gleason 3 ROIs present in each cluster (if present). Clusters are identified by color in the t-SNE projection, and all points that are not Gleason 3 (triangles and squares) are colored in grey. The Gleason 3 bar on the Gleason frequency counts is colored to match the corresponding cluster in the t-SNE projection. Averaged intensity diagrams are computed for Gleason 3 persistence diagrams exclusively, by cluster.

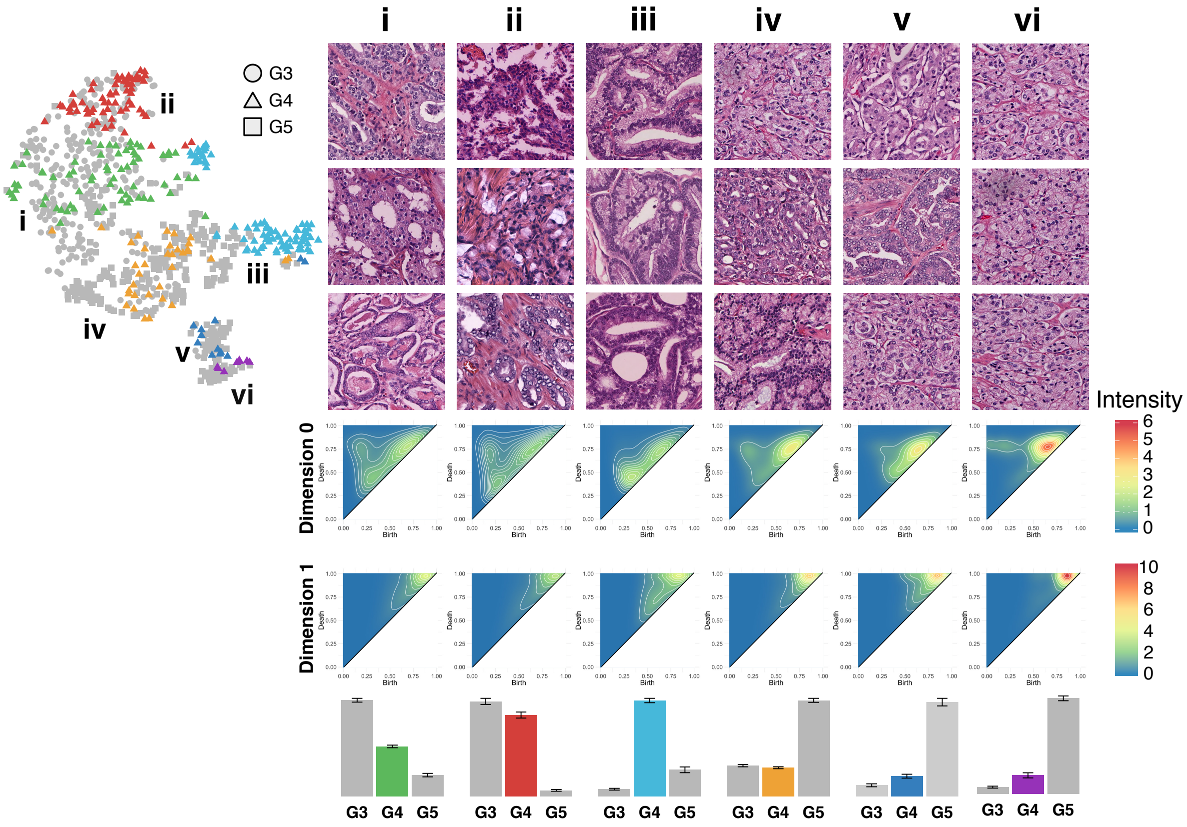

Figure 8. Comparison of Gleason 4 ROIs and intensity diagrams across all clusters. Example ROIs and averaged intensity diagrams for Gleason 4 ROIs present in each cluster (if present). Clusters are identified by color in the t-SNE projection, and all points that are not Gleason 4 (circles and squares) are colored in grey. The Gleason 4 bar on the Gleason frequency counts is colored to match the corresponding cluster in the t-SNE projection. Averaged intensity diagrams are computed for Gleason 4 persistence diagrams exclusively, by cluster.

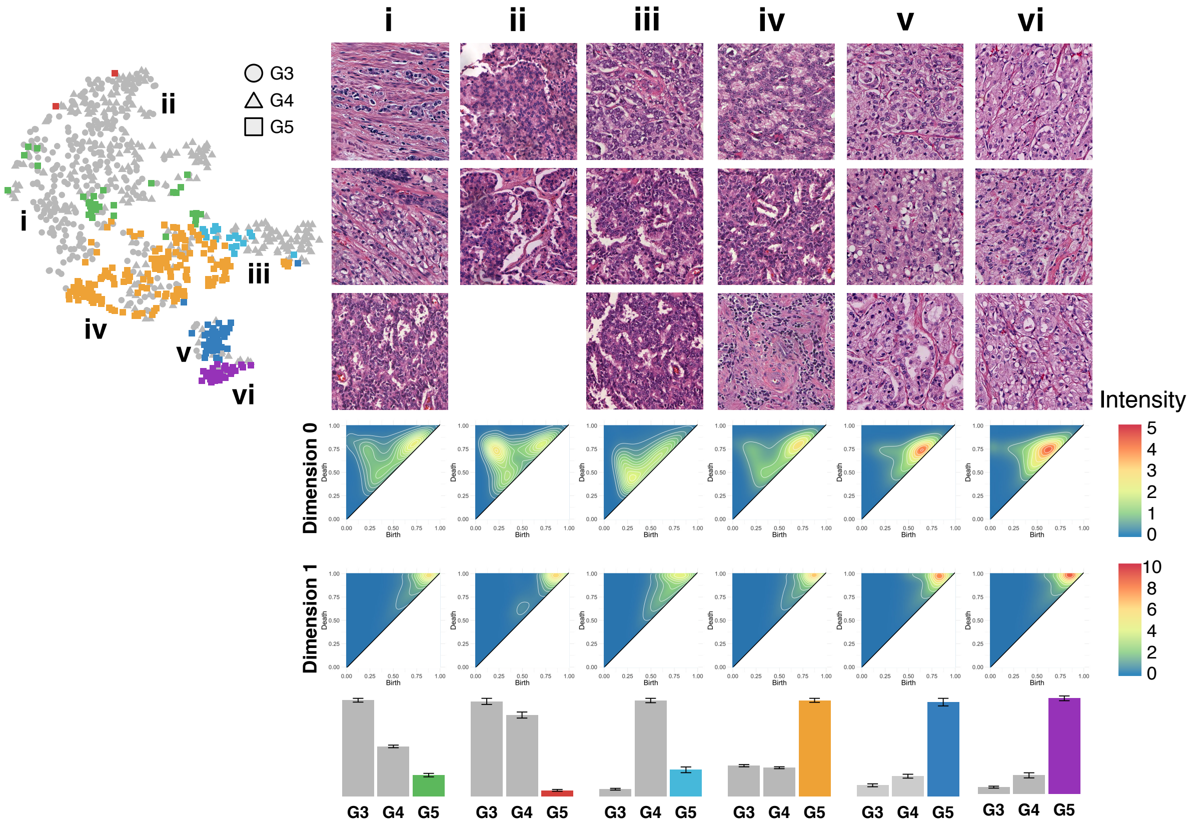

Figure 9. **Comparison of Gleason 5 ROIs and intensity diagrams across all clusters.** Example ROIs and averaged intensity diagrams for Gleason 5 ROIs present in each cluster (if present). Clusters are identified by color in the t-SNE projection, and all points that are not Gleason 5 (circles and triangles) are colored in grey. The Gleason 5 bar on the Gleason frequency counts is colored to match the corresponding cluster in the t-SNE projection. Averaged intensity diagrams are computed for Gleason 5 persistence diagrams exclusively, by cluster.
